# Beyond Diagnosis: Preliminary Study of Impact on Children and Parents in Neurodevelopmental Disorders and Juvenile Idiopathic Arthritis-Associated Uveitis

**DOI:** 10.3390/diagnostics14030275

**Published:** 2024-01-26

**Authors:** Roberta Palmieri, Valeria Albano, Silvana Guerriero, Francesco Craig, Francesco La Torre, Serena Filoni, Dario Sardella, Maria Giuseppina Petruzzelli, Paola Lecce, Andrea De Giacomo

**Affiliations:** 1 Translational Biomedicine and Neuroscience Department (DiBraiN), University of Bari “Aldo Moro”, 70124 Bari, Italy; dario.sardella@gmail.com (D.S.); maria.petruzzelli@uniba.it (M.G.P.); paolaalecce@gmail.com (P.L.); andrea.degiacomo@uniba.it (A.D.G.); 2 Department of Basic Medical Sciences, Neurosciences and Sense Organs, Institute of Ophthalmology, University of Bari, Piazza Giulio Cesare 11, 70124 Bari, Italy; valeria.albano12@gmail.com (V.A.); silvanaguerriero@gmail.com (S.G.); 3 Department of Cultures, Education and Society (DICES), University of Calabria, 87036 Cosenza, Italy; francesco.craig@unical.it; 4 Department of Pediatrics, Pediatric Rheumatology Center, “Giovanni XXIII”, Pediatric Hospital, Via Giovanni Amendola 207, 70126 Bari, Italy; latorre_francesco@virgilio.it; 5 I.R.C.C.S. Casa Sollievo della Sofferenza, 71013 San Giovanni Rotondo, Italy; serena.diba@gmail.com

**Keywords:** chronic diseases, neurodevelopmental disorders, juvenile idiopathic arthritis, uveitis, emotional problems, behavioral problems, parental stress, mental health prevention

## Abstract

Chronic diseases are a growing problem for global health due to the large number of people they involve, the repercussions they have on the mental and physical well-being of those affected, and the costs to society. Particularly, chronic illnesses of childhood have important psychological implications, not only for affected children but also for their parents. Among these pathologies, neurodevelopmental disorders (NDDs) and uveitis associated with juvenile idiopathic arthritis (JIA-U) may affect mental and physical health, emotions, memory, learning, and socializing. This study evaluates the psychological and behavioral/emotional impact of NDDs and JIA-U on children and parents. Specifically, 30 children with active JIA-U and 30 children with NDDs and their parents completed the Child Behavior Checklist (CBCL) and Parent Stress Index—Short Form (PSI) questionnaires. Children with NDDs have statistically significant differences in all the emotional and behavioral variables compared to JIA-U children, and parents of children with NDDs experience an increased stress load compared to parents of children with JIA-U. This study emphasizes the wide range of emotional and behavioral challenges that parents face with NDDs. This study emphasizes that parents of children with NDDs not only experience higher levels of stress compared to parents of normally developing children but also experience higher levels of stress compared to parents of children with potentially debilitating chronic diseases such as JIA-U.

## 1. Introduction

The World Health Organization (WHO) defines chronic disease as a condition that persists for a long period, typically at least 3–12 months, and requires continuous medical care [1].

Chronic diseases are constantly increasing, both among adults and children, and they have a significant impact on the psychophysical well-being of those affected but also on caregivers [2,3]. Furthermore, these conditions are a leading cause of disability globally, contributing to a substantial burden on healthcare systems and society [4]. They encompass a wide range of conditions, including diabetes, cancer, liver diseases, chronic obstructive pulmonary disease, chronic kidney disease, and neurological and mental health disorders [5,6,7].

Chronic childhood diseases are also on the rise, affecting 10% to 12% of children worldwide with serious consequences for affected children and their families [8]. Chronic diseases such as early childhood cystic fibrosis, psoriasis, asthma, and atopic dermatitis have been demonstrated to significantly diminish the quality of life for children who are impacted by them [9] but also very different conditions including asthma, renal disease, epilepsy, and enuresis. The negative impact of chronic diseases in childhood is also reflected in the psychological functioning of children and their mothers, as well as the emotional health of parents [10]. Multiple studies have repeatedly demonstrated that parents of children with chronic diseases endure elevated levels of stress in comparison to parents of healthy children [11].

Parental stress is defined as an adverse psychological reaction when parents perceive a disparity between the child’s request and his or her ability to cope adequately [12]. This stress is often related to the child’s illness and the additional responsibilities and challenges it brings, such as managing the child’s behavior problems [13], coping with the demands of the prescribed therapy [14], and dealing with the chronic nature of the disease [15]. The impact of chronic illness on parents is further exacerbated by factors such as family functioning, level of education, coping strategies, and resilience [16]. For instance, less-acculturated parents may experience higher chronic stress while parents with good coping strategies tend to have lower parenting stress [17]. The stress experienced by parents of children with chronic illnesses can have negative implications for both the parents and the children. It has been associated with poorer psychological adjustment in both parents and children as well as poor child adjustment outcomes such as behavior problems and poor social competence [18]. Additionally, the stress experienced by parents may vary depending on the specific chronic illness of the child but it is consistently higher than that experienced by parents of healthy children [19].

Among these chronic diseases, neurodevelopmental disorders (NDDs) are neurologic conditions that impact the development of the brain, characterized by a range of cognitive and behavioral issues. The prevalence of neurodevelopmental disorders is substantial, with an estimated 15% of individuals aged 3 to 17 years in the United States being affected [20] and with an estimated 53 million affected children and young people globally [21]. These abnormalities become evident early and are characterized by impairments in personal, academic, social, and occupational abilities [22,23]. Specifically, NDDs include Autism Spectrum Disorder (ASD), Attention Deficit Hyperactivity Disorder (ADHD), Specific Learning Disorders (SpLD), Intellectual Disability (ID), Specific Communication Disorders (LD), and Movement Disorders (MD).

The etiopathogenesis of these disorders is complex and multifaceted because it involves a combination of genetic, environmental, and neurodevelopmental factors. Several studies have highlighted the significance of prenatal factors, such as parental age, maternal diabetes [24], hypertensive disorders of pregnancy (HDP) [25], and exposure to organophosphate insecticides [26], in contributing to the etiopathogenesis of neurodevelopmental disorders. 

Moreover, the timing of exposure to immunological challenges during prenatal development has been associated with the particularity of brain and behavioral abnormalities caused by inflammation, suggesting a critical role of precise timing in the relationship between in utero infection and the development of mental disorders with a presumed developmental origin [27].

Furthermore, environmental factors, such as exposure to allergens during fetal development, have been associated with an increased incidence of neurodevelopmental disorders, including ASD [28]. Additionally, the season of birth and exposure to influenza during pregnancy have been identified as risk factors for the development of schizophrenia, emphasizing the role of environmental factors in the etiopathogenesis of neurodevelopmental disorders.

Moreover, neurodevelopmental disorders have been linked to neuroanatomical and neurochemical alterations [29].

The clinical features of neurodevelopmental disorders encompass a wide array of heterogeneous symptoms and characteristics that manifest across different conditions.

Individuals with neurodevelopmental disorders may exhibit cognitive deficits, language impairments, and motor coordination difficulties. They can also present deficits in social communication and interaction, a hallmark feature of ASD [30]. This may include challenges in understanding and using nonverbal communication, difficulties in developing and maintaining relationships, and atypical social–emotional reciprocity. Additionally, restricted and repetitive patterns of behavior, interests, or activities are common in individuals with ASD, often leading to inflexible adherence to routines and highly specific interests. Sensory dysregulation is another common symptom observed in neurodevelopmental disorders, including hypersensitivity to sensory stimuli, such as touch, sound, or light [31].

In the context of ADHD, symptoms may include inattention, hyperactivity, and impulsivity. Individuals with ADHD may struggle with sustaining attention, organizing tasks, and following through on instructions. They may also display restlessness, fidgeting, and difficulty engaging in activities quietly. Impulsivity may manifest as interrupting others, intruding on conversations, and acting without considering consequences [32].

Furthermore, individuals with neurodevelopmental disorders may experience executive dysfunction, affecting their ability to plan, organize, and execute tasks. This can lead to difficulties in time management, problem-solving, and self-regulation.

It is important to note that the symptoms of neurodevelopmental disorders often overlap, and individuals may present with a combination of symptoms from different domains. Additionally, the severity and presentation of symptoms can vary widely among individuals, highlighting the heterogeneous nature of neurodevelopmental disorders.

The diagnosis of neurodevelopmental disorders is essentially clinical; however, the incorporation of new stratification methods and clinical algorithms has been proposed to enhance surveillance, screening, evaluation, diagnosis, and management of developmental disorders in high-risk populations, potentially leading to improved neurodevelopmental and behavioral outcomes. Additionally, electrophysiological biomarkers have been identified as potential tools for the diagnosis and prognosis of neurodevelopmental disorders [33].

The treatment of neurodevelopmental disorders requires a multifaceted approach, encompassing early intervention, cognitive interventions, and targeted pharmacological treatments [34]. There are ongoing efforts to create new treatments for genetic neurodevelopmental disorders. However, there is a shortage of clinical outcome measures and biomarkers that are specifically relevant to these diseases [35]. Research into the efficacy of nutrition-related interventions for treating neurodevelopmental disorders is increasing [36]. Additionally, the use of transcutaneous auricular vagus nerve stimulation is being explored as a potential therapeutic application in neurodevelopmental and other pediatric disorders [37].

Early intervention is crucial for supporting the development of individuals with neurodevelopmental disorders [38]. 

For all these reasons and the complexity of the clinical picture, parents of children with NDDs show higher levels of parental stress compared to parents of typically developing children due to the commitment related to the management of their children [39].

Another very common chronic disease is juvenile idiopathic arthritis (JIA), which is a chronic condition characterized by persistent joint inflammation for at least 6 weeks, with an onset before the age of 16 years and an unknown cause [40], and it is the commonest rheumatic disease in children [41]. The symptoms of JIA can vary widely but commonly include persistent joint pain, swelling, and stiffness, which can lead to functional impairment and reduced quality of life [42]. The etiopathogenesis of JIA is complex and multifactorial, with environmental factors being considered relatively controllable determinants [43]. The pathogenesis of JIA involves the dysregulation of immune responses, with the involvement of various immune cells and proinflammatory cytokines such as Th17 and Th1 lymphocytes, tumor necrosis factor-alpha, and interleukin-1 [40,44,45].

The diagnosis of JIA is based on clinical criteria, and additional methods such as ultrasound and radiological imaging are often required to detect typical features of the disease [46]. Additionally, JIA is associated with an increased risk of certain complications, such as chronic uveitis, gastrointestinal tract changes, fever, rashes, systemic inflammation, and bone erosions [47,48]. Uveitis-associated juvenile idiopathic arthritis (JIA-U) is its most frequently an extra-articular manifestation, accounting for 47% of all types of uveitis in children [49], and is a common and devastating complication of JIA, with a high incidence and potential for permanent vision loss. The risk of developing uveitis is greatest in the first four years after the onset of arthritis (82–90%) [50].

JIA-U is categorized according to the specific ocular compartment that is affected (anterior, intermediate, posterior, or panuveitis) and the temporal pattern of inflammation (acute, subacute, chronic, or recurring). Among children with JIA-U, the most common form is the chronic anterior and bilateral kind [51]. Significantly, JIA-U often results in serious problems, such as irreversible visual loss, particularly when not addressed [52].

The ocular involvement is asymptomatic most frequently [53]; therefore, the short and medium–long term complications arising from JIA-U—such as visual loss, chronic therapy (topical systemic or biological molecular), trend of remission, and recrudescence—have strong psychological implications on the children and their families.

However, only a limited number of studies have investigated the psychological consequences of JIA-U [54,55], with the majority focusing on adult patients [56,57] and only a few studies investigating children and their parents [58].

The objective of this study is to assess and compare the impact that chronic conditions such as NDDs and JIA-U have on children’s mental health in terms of emotional and behavioral problems. Moreover, this research aims to assess parental stress related to the management of these conditions.

## 2. Materials and Methods

### 2.1. Participants

This study was a retrospective analysis that involved a cohort of children diagnosed with oligoarticular JIA-U and their parents, as well as a cohort of children diagnosed with NDDs and their parents. Particularly, the uveitis group included 30 children (13 males and 17 females, mean age was 9.77 ± 3.57 years) with active JIA-U, in accordance with the International League of Associations for Rheumatology (ILAR) criteria, referred to the Unit of Ophthalmology, Giovanni XXIII Hospital of Bari with their parents. Enrolled subjects underwent ophthalmologic examination, during which standardized questionnaires were administered. Questionnaires were completed by 26 mothers and by 4 fathers. The study enrolled children who displayed anterior chamber cells, anterior chamber flare, vitreous cells, haze, or retinal edema, as per the standardization of uveitis nomenclature (SUN) criteria. The exclusion criteria encompassed any systemic or ocular conditions that had the potential to impact vision. The NDD group included 30 children (20 males and 10 females, mean age 8.67 ± 4.00 years) referred to the Child Neuropsychiatry Unit Polyclinic of Bari. Specifically, the group included 6 children diagnosed with ASD, 7 subjects with ID, 6 children with LD, 5 subjects with SpLD, and 6 children with ADHD. The enrolled participants received a diagnosis of NDD according to criteria defined by the Diagnostic and Statistical Manual of Mental Disorders–5th ed. (DSM-5). Questionnaires were completed by 28 mothers and by 2 fathers during neuropsychiatric evaluation of the children. Exclusion criteria were disabling neurological conditions and psychiatric disorders in the parents. Data were recorded from March to December 2020, during the first lockdown imposed by Italian government during the COVID-19 pandemic. All parents signed informed consent.

Table 1 provides a summary of the socio-demographic features of both the JIA-U group and the NDD group.

### 2.2. Assessment

The evaluation was conducted by administering standardized scales, the Child Behavior Checklist (CBCL) [59] and the Parental Stress Index—Short Form (PSI-SF) [60], in both study groups.

The CBCL is a parent-reported evaluation that uses questionnaires to evaluate a child’s behavioral and emotional challenges. There is a preschool version consisting of 99 items designed for children between the ages of 1.5 years and 5 years [61]. Additionally, there is a school-aged version consisting of 113 items intended for children between the ages of 6 and 18 years [62]. The items in question are designed to assess particular emotional and behavioral issues. They are evaluated using a three-point Likert scale, ranging from 0 (indicating the absence of the issue) to 2 (indicating a high frequency of occurrence). Scoring is performed for syndrome scales, DSM-oriented scales, composite clinical scales (internalizing and externalizing), and one overall clinical scale. T-scores are derived from raw scores. T-scores of 70 or higher on the syndrome and DSM-oriented scales, and 64 or higher on the composite and total scales, suggest clinically significant elevations. T-scores ranging from 65 to 69 on the symptom and DSM-oriented scales, and 60 to 63 on the composite and total scales, indicating borderline or subclinical increases. Lavigne et al. [63] reported that the CBCL had an average sensitivity of 0.63 and an average specificity of 0.84.

The PSI-SF is a self-administered survey derived from the comprehensive 120-item version. It assesses the level of stress experienced by parents in their capacity as caregivers. The PSI-SF consists of 36 items that are divided into 3 subscales, with each subscale containing 12 items. These subscales are parental distress (PD), dysfunctional parental–child interaction (D-PCI), and challenging child (DC). PD (items 1–12) measures stress associated with parent traits, such as feelings of competence, spousal conflict, social support, limitations, and parental depression. D-PCI (items 13–24) assesses parental contentment over their child and the quality of their relationship with them. A high score in this domain indicates that the parent views their child as not meeting their expectations and that their interactions with the child do not strengthen their role as a parent. DC (items 25–36) assesses the level of challenge parents face in caring for their child primarily as a result of the child’s behavioral traits. The test incorporates a Defensive Responding (DF) scale that assesses the extent to which the parent tends to downplay or underestimate the problems. Consequently, it is anticipated that parents of children with neurodevelopmental disorders (NNDs) will express higher levels of stress in this particular area. The PSI-SF includes a PSI total subscale, which is the sum of all scores, providing a quantification of an individual’s overall stress levels as a parent. The majority of the items (33) are evaluated using a 5-point Likert scale, ranging from 1 (indicating severe disagreement) to 5 (indicating strong agreement). Conversely, three items (22, 32, 33) lack a Likert-type response option.

### 2.3. Statistical Analysis

Descriptive statistics (i.e., mean values and standard deviations) were used to summarize the variables studied and the characteristics of the subjects. Since the data distribution was not normal (evaluated through Shapiro–Wilk test), non-parametric tests (Mann–Whitney) were used to examine the difference in CBCL and PSI-SF items between the groups. A *p*-value of less than 0.05 was considered statistically significant. For statistical processing, we used the Statistical Package for Social Science (IBM SPSS, version 27) data processing program.

## 3. Results

The statistical analysis of the emotional and behavioral problems shows significant statistical differences between the two groups for all variables of the CBCL questionnaire, as shown in Table 2.

The NDD group reported higher scores than the JIA-U group in total problems (*p* < 0.001). About the externalizing problems, the NDD group reported higher values in attention problems (*p* < 0.001), aggressive behavior (*p* < 0.001), ADHD problems (*p* < 0.001), and oppositional defiant problems (*p* < 0.001). Instead, regarding internalizing problems, the NDD group showed higher values in anxious/depressed (*p* < 0.001), withdrawn (*p* < 0.001), somatic complaints (*p* < 0.001), affective problems (*p* < 0.001), and anxiety problems (*p* < 0.001).

**Figure 1:**
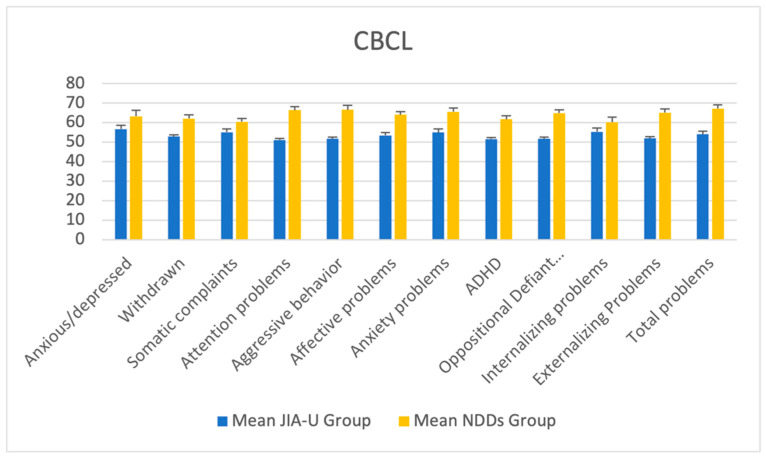
Mean values of CBCL T-scores and standard errors for the NDD and JIA-U groups.

The mean values of the CBCL T-scores and standard errors for the NDD and JIA-U groups are represented in Figure 1.

The measure collected to assess differences in parenting stress reveals statistically significant variations in all variables of the PSI-SF questionnaire, as reported in Table 3.

Specifically, the NDD group reported higher scores than JIA-U parents in PD (*p* < 0.001), P-CDI (*p* < 0.001), P-DC (*p* < 0.001), P-DF (*p* < 0.001), and total stress (*p* < 0.001).

## 4. Discussion

The objective of this study is to assess the influence of chronic diseases, such as NDD and JIA-U, on the mental well-being of children in terms of behavioral and emotional difficulties, as well as the impact on their parents.

Indeed, these illnesses are distinguished by their enduring nature, which has a substantial influence on the psychological well-being of those affected as well as their caregivers. To the best of the authors’ knowledge, the present work is the first to compare psychological implications on children and parental stress of NDDs and JIA-U.

In particular, the results of this study show major psychological implications regarding emotional and behavioral problems of NDDs than JIA-U and that, at the same time, the parents of these children are also subjected to a greater share of stress, with a significant difference in all the variables involved.

These findings are in agreement with previous studies. In fact, it is well-known that NDDs are associated with high rates of behavioral and emotional problems such as social–communication deficits, repetitive and stereotyped comportment, hyperactivity, food selectivity, motor deficits, cognitive impairment, anxiety, self-injury, disruptive behaviors, and sleep disorders [64,65].

Behavioral/emotional problems are an intrinsic feature of NDDs; however, this study shows that their extent in affected children is greater not only than in typically developing children but also in children with chronic diseases such as JIA-U.

The results related to parental stress reveal that the management of NDDs is more impacting when compared to the management of JIA-U, despite the chronic and disabling nature of this pathology.

Research consistently indicates that parents of children with NDDs frequently report elevated levels of stress, anxiety, sadness, and diminished quality of life in comparison to parents of generally developing children [23,39,66,67,68,69,70,71,72,73].

This stress is particularly pronounced among mothers of children with ASD and other neurodevelopmental disabilities [66,68,71,74].

Parental stress is multifaceted, influenced by various factors such as the nature of the illness, the severity of symptoms, family functioning, coping strategies, and acculturation.

Parents experiencing significant levels of stress exhibit reduced capacity to effectively execute interventions for their children with impairments, resulting in diminished developmental advancements in their children.

Moreover, the COVID-19 pandemic has intensified the levels of stress experienced by parents of children with neurodevelopmental disorders (NDDs) due to the loss of continuity of care and the impact on the well-being of children with NDDs [23,72].

However, in agreement with our findings, the comparison of parenting stress between NDDs and other pediatric diseases consistently demonstrated that parents of children with NDDs experience more stress, illness, and psychiatric problems compared to parents of children with other disabilities or chronic conditions [66,69,70,75]. Additionally, challenging child behaviors in children with NDDs positively predict symptoms of posttraumatic stress disorder in parents, further highlighting the significant impact of parenting stress in this population [73].

Previous studies have clearly shown that parents of children with NDDs feel significantly higher levels of stress compared to parents of children with epilepsy [76]. Prior research indicates that parents of children with NDDs experience a higher level of stress compared to parents of children with epilepsy, even though epilepsy itself is already recognized as a source of stress. This implies that the difficulties related to looking after children with autism may have a greater influence on parental stress in comparison to caring for children with epilepsy.

Parents of autistic children exhibit higher levels of stress compared to parents of children with other disorders.

Hayes and Watson [77] performed a meta-analysis, which revealed that families of children with ASD encounter higher levels of parenting stress compared to families of typically developing children or those with other disabilities including cerebral palsy, Down Syndrome, and sensory disorders (blindness, visually impaired, deaf, hard of hearing). Furthermore, Scherer et al. [78] conducted a systematic review and meta-analysis where 69% of the research specifically targeted parents of children diagnosed with cerebral palsy or autism, highlighting the impact of these conditions on parental stress. Pan et al. [79] also conducted a systematic review and meta-analysis, focusing on neurological disorders in autism, with seven case-control studies focusing on cerebral palsy in autism. Notably, although cerebral palsy could be accompanied by several motor and cognitive disabilities [80,81,82], these studies consistently confirm the idea that parents of children with autism encounter elevated levels of stress in comparison to parents of children with cerebral palsy and other disorders.

Parents of children with ASD and ADHD exhibited higher levels of parental stress in comparison to parents of children and adolescents with anxiety disorder [83].

There are constraints to take into account while analyzing the findings of this study. Firstly, the sample size is small, and replication with larger samples is needed. Furthermore, the NDD group is heterogeneous, encompassing all neurodevelopmental disorders. It will be useful to compare the psychological impact and parenting stress of JLA-U with less heterogeneous groups to better understand possible differences with respect to each individual neurodevelopmental disorder. Moreover, it is important to include a major number of fathers, as their perspectives may differ from that of mothers. Finally, in interpreting these results, it is necessary to consider the strong disparity of treatment options of JIA-U compared to NDDs. This will include the use of conventional immunosuppressants, such as glucocorticoids and methotrexate, biologic anti-tumor necrosis factor agents, such as adalimumab, as well as other anti-tumor necrosis factor agents, including infliximab and golimumab and medications currently in clinical trials including interleukin-6 inhibitors (tocilizumab) and Janus kinase inhibitors (tofacitinib, baricitinib) [50]. These treatments aim to control inflammation and minimize the risk of sight-threatening complications associated with chronic uveitis in JIA. On the other hand, the scope for intervention for NDDs is much smaller, despite the efforts of the scientific community in identifying the causes and specific treatments for these conditions.

In conclusion, chronic childhood diseases such as NDDs and JIA-U have a substantial and multifaceted negative impact on affected children and their families. This impact encompasses physical, psychological, and social aspects of well-being and can persist into adulthood. In particular, the core symptoms of NDDs seem to have a greater impact on children’s psychophysical well-being and parental stress than even a disabling pathology such as JIA-U.

This underlines the importance of early management of children with NDDs also including coping strategies and interventions to support parents. These include the use of coping health inventories and parental stress scales to assess coping strategies and stress levels [17] as well as the implementation of group programs aimed at providing families with pertinent knowledge and new coping skills in a psychoeducational setting [84]. Moreover, filial therapy has been proposed as a possible intervention to assist parents in managing the stress linked to their child’s persistent sickness [85].

## 5. Conclusions

This study compared the stress and behavioral/emotional conditions of children with NDDs and JIA-U and their parents. The results demonstrated that parenting stress is significantly higher among parents of children with NDDs compared to parents of JIA-U. This highlights the need for targeted support and interventions to address the unique challenges faced by parents of children with NDDs.

## Figures and Tables

**Table 1:** Social demographic characteristics of patients and their parents in the two groups.

	JIA-U Group	NDD Group
Number	30	30
Age (years)	9.77 ± 3.57	8.67 ± 4.00
Gender		
Male	13	20
Female	17	10
Mother (*n*)	26	28
Father (*n*)	4	2

**Table 2:** Differences in scores between groups found in CBCL.

	JIA-U Group	NDD Group	Mann–Whitney U	Z	*p*-Value
Anxious/depressed	56.73 ± 9.95	63.27 ± 16.52	260.50	−2.913	0.004
Withdrawn	52.87 ± 5.32	62.10 ± 10.63	161.50	−4.421	<0.001
Somatic complaints	55.10 ± 9.59	60.50 ± 8.50	219.50	−3.528	<0.001
Attention problems	51.17 ± 4.09	66.37 ± 10.16	57.00	−6.172	<0.001
Aggressive behavior	51.73 ± 4.29	66.63 ± 12.96	95.00	−5.541	<0.001
Affective problems	53.50 ± 7.71	64.07 ± 8.95	157.00	−4.577	<0.001
Anxiety problems	55.00 ± 9.54	65.57 ± 10.40	162.00	−4.446	<0.001
ADHD	51.67 ± 5.14	64.93 ± 8.76	62.00	−6.024	<0.001
Oppositional defiant problems	51.57 ± 4.26	61.73 ± 10.12	122.50	−5.148	<0.001
Internalizing problems	55.37± 9.93	60.23 ± 14.82	299.50	−2.301	0.021
Externalizing Problems	52.00 ± 4.28	65.10 ± 10.91	114.50	−5.179	<0.001
Total problems	54.07 ± 8.37	67.23 ± 11.09	148.50	−4.631	<0.001

**Table 3:** Differences in scores between groups found in PSI-SF.

	JIA-U Group	NDD Group	Mann–Whitney U	Z	*p*-Value
Parenting distress	24.90 ± 7.43	35.60 ± 8.33	150.00	−4.446	<0.001
Dysfunctional interaction parent–child	21.50 ± 6.28	30.53 ± 4.80	119.5	−4.894	<0.001
Difficult child	24.77 ± 8.07	37.17 ± 7.87	119.5	−4.894	<0.001
Defensive responding	15.93 ± 5.61	21.27 ± 4.95	218.5	−3.434	<0.001
Total Stress	71.17 ± 19.51	103.30 ± 13.82	76.5	−5.524	<0.001

## Data Availability

The data presented in this study are available upon request from the corresponding author.
